# Bayesian Modeling of Cognitive Impairment in the Presence of Retest Effects

**DOI:** 10.1093/geroni/igaa057.1870

**Published:** 2020-12-16

**Authors:** Zita Oravecz, Nelson Roque, Martin Sliwinski

**Affiliations:** 1 Pennsylvania State University, State College, United States; 2 Pennsylvania State University, University Park, Pennsylvania, United States; 3 Penn State University, University Park, Pennsylvania, United States

## Abstract

Diagnosing the early onset of neuropathologies, such as mild cognitive impairment (MCI), requires repeated evaluation of cognitive skills several times per year -- a measurement design known as a “burst design.” Detecting the often subtle cognitive decline in the presence of retest effects requires careful statistical modeling. The double exponential model offers a modeling framework to account for retest gains across measurement bursts, as well as warm-up effects within a burst, while quantifying change across bursts in peak performance. This talk highlights how a Bayesian multilevel implementation of the double exponential model allows for flexible extensions of this framework in terms of accommodating different timescales (nesting) and person-level predictors and drawing intuitive inferences on cognitive change with Bayesian posterior probabilities. We will use reaction time data to show how individual differences in asymptotic performance and change can be related to predictors such as age and MCI status. Part of a symposium sponsored by the Measurement, Statistics, and Research Design Interest Group.

